# A meta-analysis of the diagnostic accuracy of dual-energy computed tomography for endoleak detection after endovascular aneurysm repair

**DOI:** 10.1007/s00330-025-11717-8

**Published:** 2025-06-07

**Authors:** Cynthia Xin Wen, Shivshankar Thanigaimani, Sonja Brennan, Joseph Moxon, Jonathan Golledge

**Affiliations:** 1 Queensland Research Centre for Peripheral Vascular Disease, College of Medicine and Dentistry, James Cook University, Townsville, QLD Australia; 2 Australian Institute of Tropical Health and Medicine, James Cook University, Townsville, QLD Australia; 3 Medical Imaging Department, Townsville University Hospital, Townsville, QLD Australia; 4 Nth Qld Maternal Foetal Medicine Unit, Townsville University Hospital, Townsville, QLD Australia; 5 Department of Vascular and Endovascular Surgery, Townsville University Hospital, Townsville, QLD Australia

**Keywords:** Dual energy CT, Endovascular repair of aortic aneurysm, Diagnostic accuracy, Radiation dose

## Abstract

**Background:**

Patients who have undergone endovascular repair of an abdominal or thoracic aortic aneurysm (EVAR or TEVAR) are recommended to undergo lifelong imaging surveillance to detect endoleaks. Gold standard imaging is by triphasic single energy computed tomography (SECT), however, dual-energy CT (DECT) can reduce radiation exposure by reducing the number of scan phases required. The accuracy of DECT in detecting endoleaks is uncertain. This review assessed the diagnostic accuracy of DECT for detecting endoleaks and compared radiation exposure to conventional triphasic SECT.

**Methods and results:**

Observational studies from Scopus and PubMed databases were screened up to 12th June 2024. Ten studies reporting the diagnostic accuracy of patients previously treated by EVAR or TEVAR were included. According to the quality assessment tool for diagnostic accuracy studies (QUADAS-2), all included studies were considered to have a high risk of bias. A meta-analysis of these studies involving 803 scans on 744 patients found that the pooled sensitivity, specificity and diagnostic odds ratio (DOR) of the DECT as compared to the triphasic SECT were 94.0% (95% CI: 88.9%, 96.9%), 98.9% (95% CI: 95.7%, 99.7%), and 266.4 (95% CI: 140.9, 503.8), respectively. A radiation dose reduction by 29–62% was achieved with DECT as compared to the triphasic SECT.

**Conclusions:**

This meta-analysis suggests that DECT has similar diagnostic accuracy to triphasic SECT for diagnosing endoleak after endovascular aneurysm repair. This suggests that DECT could be adopted in clinical practice because of the lower radiation dose as compared to the conventional triphasic SECT.

**Key Points:**

***Question*** *What is the accuracy of dual energy (DE) as compared to triphasic single energy (SE) computed tomography (CT) for diagnosing endoleak after endovascular aneurysm repair*?

***Findings*** *As compared to triphasic SECT, DECT was accurate in diagnosing endoleak after endovascular aneurysm repair and exposed patients to a lower radiation dose*.

***Clinical relevance*** *The radiation dose associated with lifelong follow-up CT scans after endovascular aneurysm repair has been linked to an increased risk of cancer. This study suggests that the use of DECT can reduce radiation exposure without compromising the accuracy of diagnosing endoleak*.

**Graphical Abstract:**

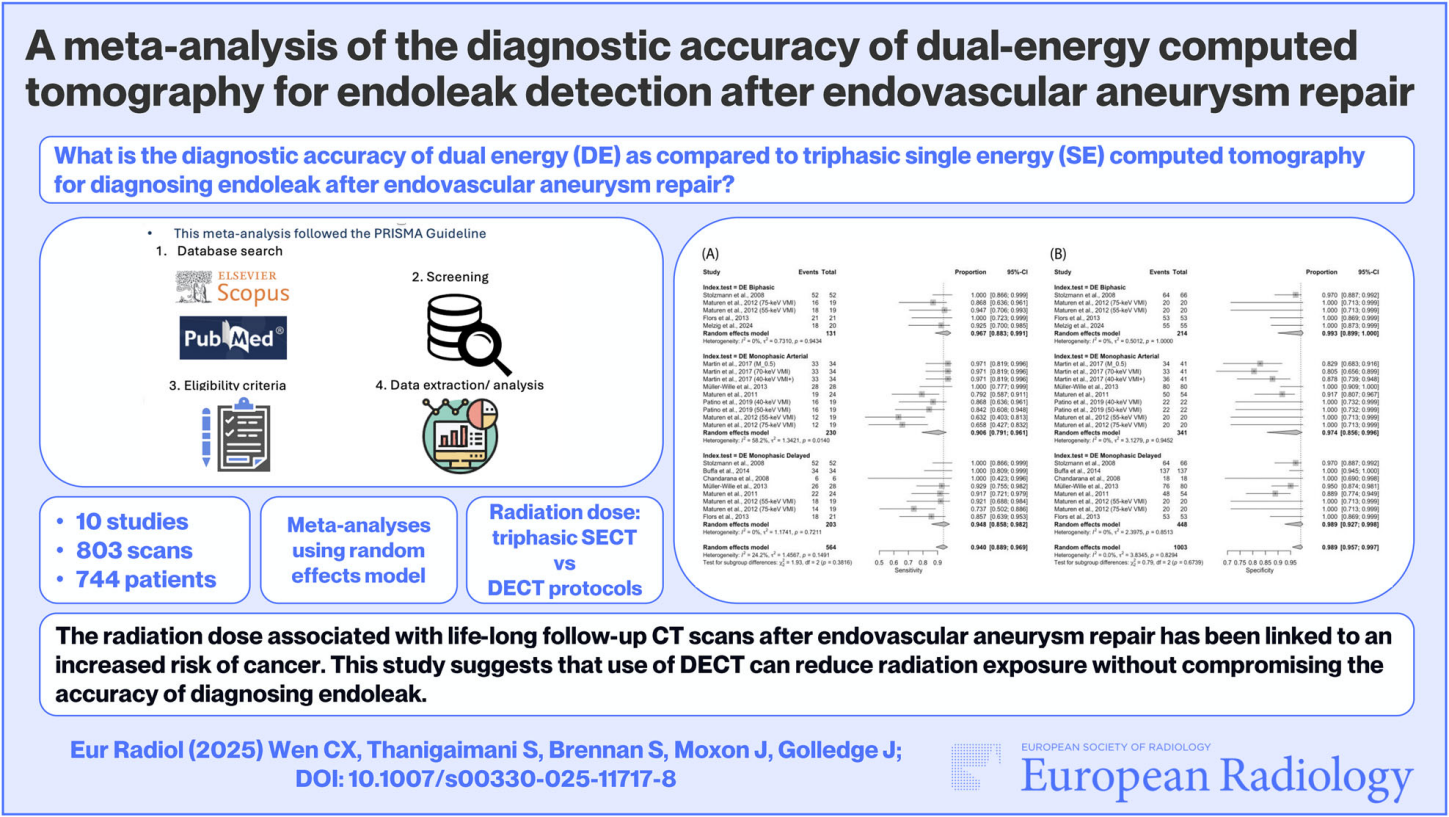

## Introduction

Aortic aneurysms, a pathological dilation of the aorta, are an important cause of cardiovascular mortality [1, 2]. Endovascular aneurysm repair, including endovascular abdominal aortic aneurysm repair (EVAR) and endovascular thoracic aneurysm repair (TEVAR), is currently the most frequently performed surgical treatment for this cardiovascular condition [1, 3–6]. The long-term outcome of this procedure is undermined by failure to adequately exclude the aneurysm sac due to endoleak, with ongoing risk of aortic rupture and death [7–11].

The European Society for Vascular Surgery and the Society for Vascular Surgery clinical practice guidelines for aortic aneurysm recommend lifelong imaging surveillance after endovascular repair with computed tomography (CT) [1, 4, 12, 13]. Currently, the gold standard for surveillance imaging is a triphasic CT scan performed under the single energy (SE) mode, which consists of a true non-contrast (TNC) phase, an arterial phase, and a delayed phase, a protocol that exposes the patient to ionizing radiation three times [4, 12]. The major disadvantage of this follow-up regime is the high cumulative radiation dose [14, 15]. The radiation exposure associated with lifelong follow-up CT scans after endovascular repair has been linked to an increased risk of abdominal and pelvic organ cancer [14, 16].

Dual energy computed tomography (DECT) involves the acquisition of two datasets from the same anatomic location with two distinctive photon spectra [17]. This technology is offering new possibilities for radiation dose reduction for these multi-phase surveillance scans. With unique DE reconstruction techniques, the TNC phase and potentially one of the contrast-enhanced phases of the conventional triphasic protocol can be safely eliminated without compromising the diagnostic accuracy, allowing the surveillance scan to be acquired at a significantly lower radiation dose [17–20]. While several observational studies have reported findings supporting the adoption of DECT [18, 21–29], no meta-analysis of these studies is currently available. The aims of this meta-analysis were to assess the diagnostic accuracy of DECT protocols for endoleak detection after endovascular repair of aortic aneurysm, and to compare the radiation dose to the conventional triphasic SECT scan.

## Methods

### Search strategy and eligibility criteria

This review was designed in compliance with the Preferred Reporting Items for a Systematic Review and Meta-Analysis of Diagnostic Test Accuracy (the PRISMA-DTA) statement [30]. The study protocol was developed a priori and was registered with PROSPERO (Registration ID: CRD42024487559). The literature search was conducted within the databases Scopus and PubMed on 24th April 2024 and updated again on 12th June 2024 independently by two authors (C.X.W. and S.T.). Any discrepancies were resolved by a third author (J.G.). The complete search string is shown in Supplementary Table S1.

The relevance of the articles was determined using pre-defined inclusion and exclusion criteria. To be eligible for inclusion, the study needed to adopt a test accuracy study design using the conventional triphasic SECT protocol as the standard of reference while providing sufficient diagnostic accuracy data of DECT protocols to allow for meta-analysis. Studies performed on phantoms or animal models, not examining aortic aneurysm repair, or not reporting endoleak diagnostic accuracy were excluded. Reviews, letters and case studies were also excluded.

### Study outcomes and data collection

The primary outcome was diagnostic accuracy of DECT protocols for endoleak detection after endovascular repair compared to findings of the reference standard (the triphasic SECT protocol), reported as the aggregated means of sensitivity, specificity, and diagnostic odds ratio (DOR) with 95% confidence interval (CI). The secondary outcome was the radiation dose reduction achievable by adopting the DECT protocol.

Two authors (C.X.W. and S.T.) independently extracted data from the included studies. Diagnostic accuracy data, including true positives, true negatives, false positives and false negatives, were extracted from the included studies to be used for meta-analysis. The average radiation dose (measured in mSv) of the DECT protocols and the triphasic SECT protocol was collected for statistical comparisons. When available, other data including population characteristics, study centre, medical history of participants, time from last EVAR, number of endoleaks and their classification, CT data acquisition protocols, DECT data post-processing protocol, were also collected.

### Risk of bias assessment

The risk of bias and applicability of the included studies were independently assessed by two authors (C.X.W. and S.T.) using the quality assessment tool for diagnostic accuracy studies (QUADAS-2) [31]. Studies found to have low risks for all key domains of QUADAS-2 were considered to be at low risk of bias. Studies found not to have low risk of bias for all key domains or where this was unclear, were considered to be at high risk of bias.

### Statistical analysis

R statistical package version 4.3.2 was used for all statistical analyses. The meta-analyses of the sensitivity, specificity and DOR reported with 95% CI were carried out by developing random-effects models using the ‘meta’ package [32]. A summary receiver operating characteristics (SROC) curve was generated using the ‘mada’ package [33] with area under the curve (AUC) calculated.

To further assess DECT’s ability for endoleak detection as compared to the reference standard (the triphasic SECT), the raw numbers of endoleak depicted by the triphasic SECT and the DECT biphasic and monophasic protocols were firstly normalized by dividing them by the total number detected by the reference standard, generating values that represented the proportion of endoleaks detected by each protocol. Due to the non-normal distribution of data, the paired Wilcoxon test was used to assess whether there was a statistical difference between the proportion of endoleaks detected by different DECT protocols and that of the reference standard triphasic SECT. Radiation dose (in mSv) of the triphasic SECT and the DE biphasic and monophasic protocols were initially compared using the Kruskal–Wallis test due to non-normal distribution of the datasets. Outliers that were more than 2 interquartile range (IQR) above or below the median were removed. After the normality test, the remaining datasets were compared again using a one-way ANOVA test. The Shapiro–Wilk test was used to assess whether datasets were normally distributed. Post-hoc power analyses were performed with G*Power [34] using the mean, standard deviation and the number of studies included in each group. The ‘ggplot2’ and ‘ggstatsplot’ from R software were used for data visualisation [35]. Continuous data was reported as mean ± standard deviation or median with IQR, depending on the normality of the data. A *p* value of < 0.05 was considered statistically significant.

## Results

### Search results

**Fig. 1:**
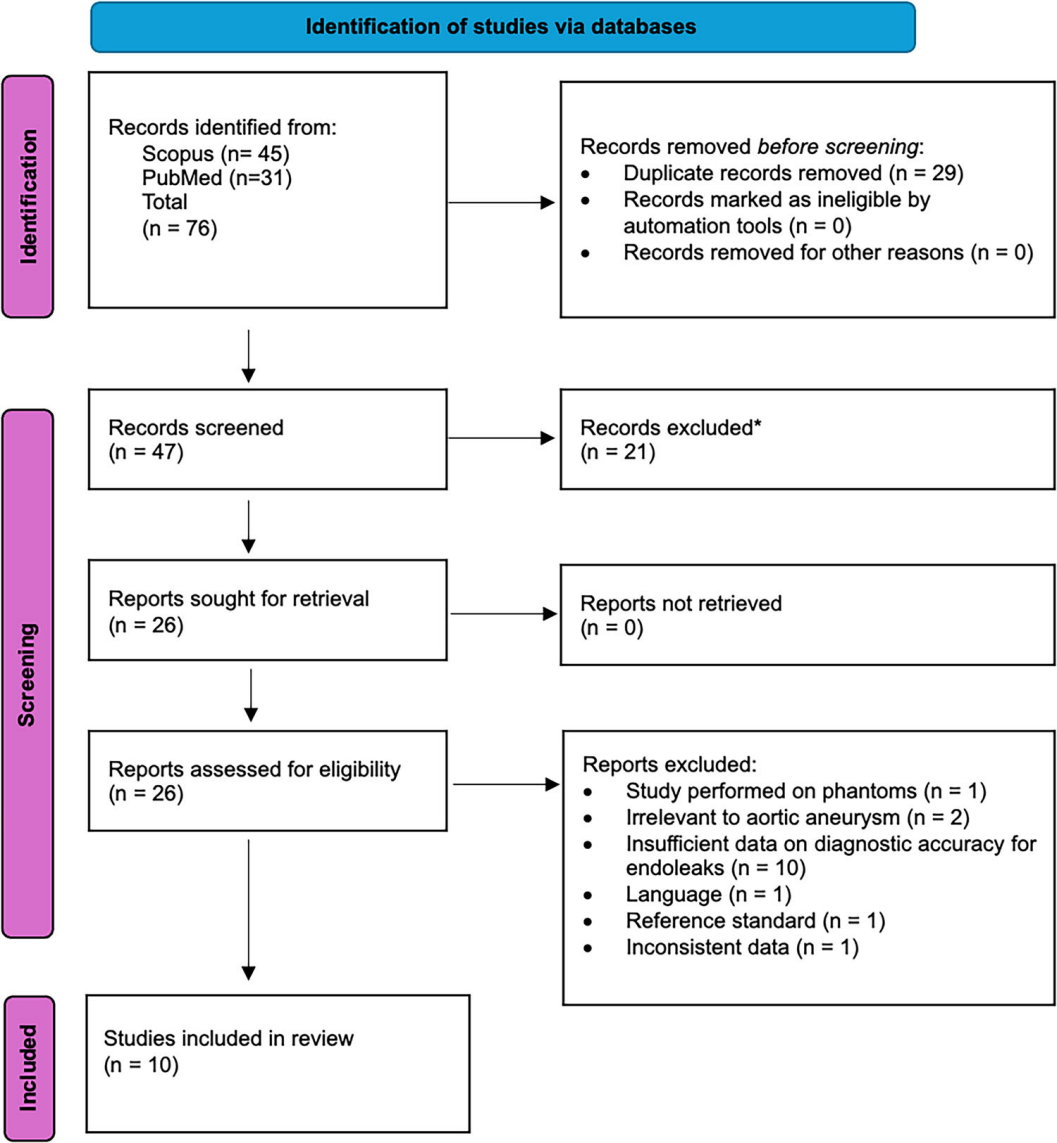
Prisma flow chart of studies included in the meta-analysis. ^*^ Reviews, letters, surveys, and book chapters are excluded at this stage

A total of 76 studies were identified. After preliminary and full text screening, a total of ten studies were eligible for inclusion [18, 21–29] (Fig. 1).

### Study characteristics

The ten studies included in this review were published between 2008 and 2024. All were single-centre studies. Six studies used a prospective design [18, 21, 22, 27–29], while four collected data retrospectively [23–26] (Supplementary Table S2).

A total of 803 CT scans from 744 patients were reported in the included studies (Supplementary Table S2). None of the studies reported patients’ comorbidities. Approximately 70% of the DECT scans included in this study were performed with dual-source dual-energy CT scanners, and the rest were obtained with fast-kilovoltage-switching DECT (Supplementary Table S3).

In these studies, the diagnostic accuracy of DECT protocols was compared against the reference standard triphasic SECT protocol (Supplementary Table S4). Two main types of DECT protocols were investigated by the included studies. The biphasic DECT protocol was made up of an arterial and a delayed phase acquisition, with one or both of these performed under the DE mode and the virtual non-contrast (VNC) images reconstructed from the DE dataset [21, 24, 25, 28]. The monophasic DECT protocol contained a DE arterial or delayed phase scan with reconstructed VNC datasets [18, 21–27, 29]. The usefulness of different DECT reconstructions, including VNC, linear-blended images, iodine overlay, virtual monoenergetic images (VMIs) and Hard Plaque Imaging, was also reported (Supplementary Table S5).

### Quality assessment

**Fig. 2:**
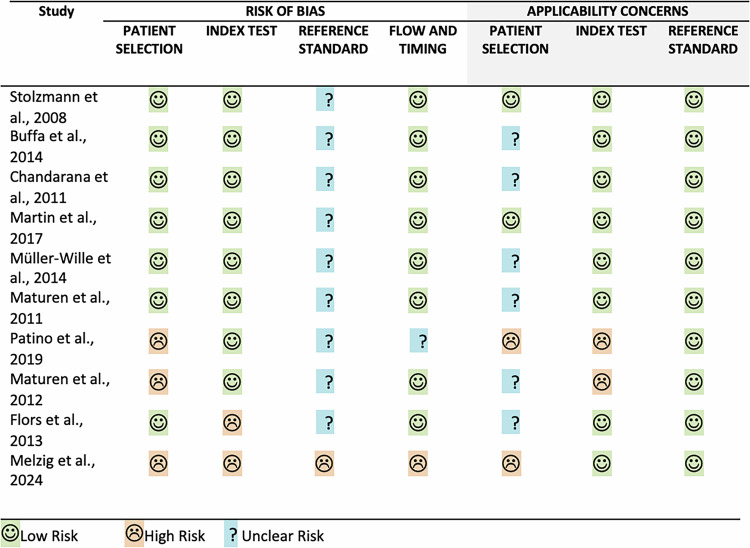
Tabular representation of risk of bias and applicability concerns for individual included studies assessed with the QUADAS-2 tool. Studies found to have low risks for all key domains of QUADAS-2 were considered to be at low risk of bias. Studies found not to have low risk of bias for all key domains or where this was unclear, were considered to be at high risk of bias. Overall, all ten studies included in this review were considered to be at high risk of bias

All ten studies were considered to be at high risk of bias [18, 21–29] (Fig. 2). For patient selection, three studies were considered to carry high risk. In one of these studies, a non-blinded author selected 40 scans out of the 108 scans available without stating the inclusion and exclusion criteria [25]. The other two studies only included patients with a previous surveillance CT scan [27, 28].

For the index test domain, two studies were deemed to be of high risk. In one of these studies, the radiologists were not blinded to the endoleaks identified in previously performed triphasic SECT [28], and the other study failed to incorporate time gaps between the reading sessions [24].

For the domain of reference standard, all ten studies displayed risk of bias [18, 21–29]. Instead of using true triphasic SECT scans as the reference standard, nine studies performed multiphase CT scans consisting of a TNC phase and contrast-enhanced phases acquired under the DE mode, with the DE dataset later reconstructed in a way that simulates conventional SE images [18, 21–27, 29]. Although generally considered a good simulation of true SE triphasic scans, this protocol is not used clinically and thus may carry the unclear risk of bias. One study used a triphasic scan performed prior to the index test (with a median time interval of 380 days) as the reference standard [28]. Since some type II endoleaks may have resolved within that period, this study was considered to have a high risk of bias for the domain of reference standard and the domain of “flow and timing” of experimental design. Another study was also considered to carry unclear risk for the domain of “flow and timing” due to a lack of information [27].

The applicability concerns of the studies were evaluated across three domains. Regarding patient selection, two studies were marked as having considerable applicability concerns due to inappropriate exclusion of patients and scans [27, 28]. Six studies did not report the time intervals between the procedure and the surveillance scans, and thus were deemed as having unclear applicability concerns under this domain [18, 22–25, 29]. Two studies were marked as having high applicability concerns under the domain of index test [25, 27], as the reporting radiologists reviewed only the VMI reconstructions of the DE scan, which did not resemble standard radiological reporting procedure.

### Diagnostic performance of DECT for endoleak detection

**Fig. 3:**
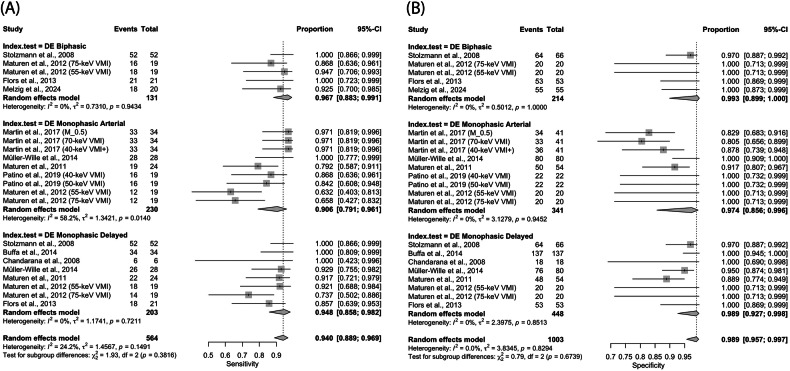
Forest plots showing meta-analysis results for sensitivity (**A**) and specificity (**B**) of DECT protocols for endoleak detection after EVAR and TEVAR using data collected from ten studies. Output from the random effects model was reported as mean sensitivity or specificity with 95% CI

**Fig. 4:**
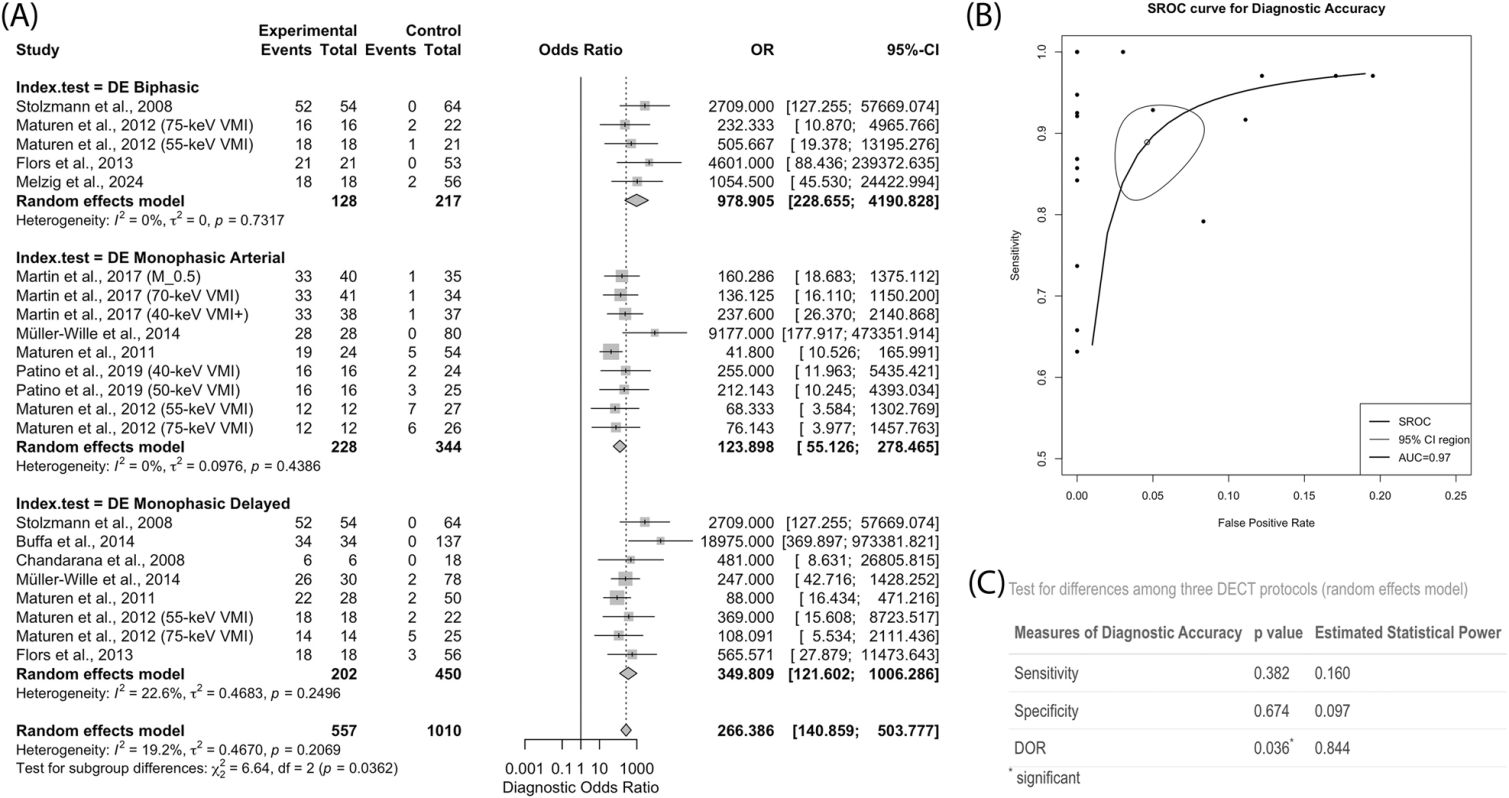
Results of further analyses by the random effects model. **A** A forest plot showing aggregated DOR for endoleak detection with DECT plotted with 95% CI. **B** An SROC curve plotted with 95% CI region and data points representing sensitivity and false positive rate (1-specificity) generated from each individual study. The AUC of the SROC curve was 0.97. **C** A table showing results from subgroup analyses for differences in aggregated sensitivity, specificity and DOR among three different DECT protocols (the DE biphasic, monophasic arterial and monophasic delayed protocols) and the estimated statistical power of each test. A *p* value of less than 0.05 was considered significant

The meta-analysis of diagnostic accuracy data from ten studies (Table 1) demonstrated that the pooled sensitivity, specificity and DOR of DECT protocols for endoleak detection were 94.0% (95% CI: 88.9%, 96.9%), 98.9% (95% CI: 95.7%, 99.7%), and 266.4 (95% CI: 140.9, 503.8), respectively (Figs. 3 and 4A). The AUC of the SROC was 0.970 (Fig. 4B). No significant inter-study heterogeneity was observed for sensitivity (*p* = 0.149, *I*^2^ = 24.2%, and τ^2^ = 1.457) (Fig. 3A), specificity (*p* = 0.829, *I*^2^ = 0.0%, and τ^2^ = 3.835) (Fig. 3B) and DOR (*p* = 0.207, *I*^2^ = 19.2% and τ^2^ = 0.467) (Fig. 4A).

**Table 1:** Diagnostic accuracy data of DECT protocols reported by included studies

Study	DECT protocols	*N*	TP	FP	FN	TN
Stolzmann et al [21]	DE biphasic	118	52	2	0	64
Maturen et al [25] (75-keV VMI)	DE biphasic	39	16.5^¤^	0	2.5^¤^	20
Maturen et al [25] (55-keV VMI)	DE biphasic	39	18	0	1	20
Flors et al [24]	DE biphasic	74	21	0	0	53
Melzig et al [28]	DE biphasic	75	18.5^¤^	0	1.5^¤^	55
Martin et al [26] (M_0.5)	DE monophasic arterial	75	33	7	1	34
Martin et al [26] (70-keV VMI)	DE monophasic arterial	75	33	8	1	33
Martin et al [26] (40-keV VMI+)	DE monophasic arterial	75	33	5	1	36
Müller-Wille et al [18]	DE monophasic arterial	108	28	0	0	80
Maturen et al [23]	DE monophasic arterial	78	19	4.5^¤^	5	49.5^¤^
Patino et al [27] (40-keV VMI)	DE monophasic arterial	41	16.5^¤^	0	2.5^¤^	22
Patino et al [27] (50-keV VMI)	DE monophasic arterial	41	16	0	3	22
Maturen et al [25] (55-keV VMI)	DE monophasic arterial	39	12	0	7	20
Maturen et al [25] (75-keV VMI)	DE monophasic arterial	39	12.5^¤^	0	6.5^¤^	20
Stolzmann et al [21]	DE monophasic delayed	118	52	2	0	64
Buffa et al [22]	DE monophasic delayed	171	34	0	0	137
Chandarana et al [29]	DE monophasic delayed	24	6	0	0	18
Müller-Wille et al [18]	DE monophasic delayed	108	26	4	2	76
Maturen et al [23]	DE monophasic delayed	78	22	6	2	48
Maturen et al [25] (55-keV VMI)	DE monophasic delayed	39	17.5^¤^	0	1.5^¤^	20
Maturen et al [25] (75-keV VMI)	DE monophasic delayed	39	14	0	5	20
Flors et al [24]	DE monophasic delayed	74	18	0	3	53

*DECT* dual-energy computed tomography, *N* number of scans, *TP*  true positive, *FP* false positive, *FN* false negative, *TN* true negative

^¤^ Decimal numerals were due to the averaging of diagnostic accuracy results from two independent readers

Subgroup analyses with the random effects model revealed no statistically significant difference in the pooled sensitivity (*p* = 0.382) and specificity (*p* = 0.674) among the three DECT protocols, namely the DE biphasic protocol, the DE monophasic arterial protocol, and the DE monophasic delayed protocol (Fig. 4C). With an estimated statistical power of 16% and 9.7%, the reliability of these two inter-protocol comparisons was markedly limited by the current sample size (Fig. 4C). However, the random effects model revealed a significant inter-protocol difference in DOR (*p* = 0.036) with a reasonable statistical power of 84% (Fig. 4C). The highest pooled DOR was achieved by the DE biphasic protocol at 978.9 (95% CI: 228.7, 4190.8). This was followed by the DE monophasic delayed protocol with a pooled DOR of 349.8 (95% CI: 121.6, 1006.3). The pooled DOR value for the DE monophasic arterial protocol was the lowest, estimated to be at 123.9 (95% CI: 55.1, 278.5) (Fig. 4A).

**Fig. 5:**
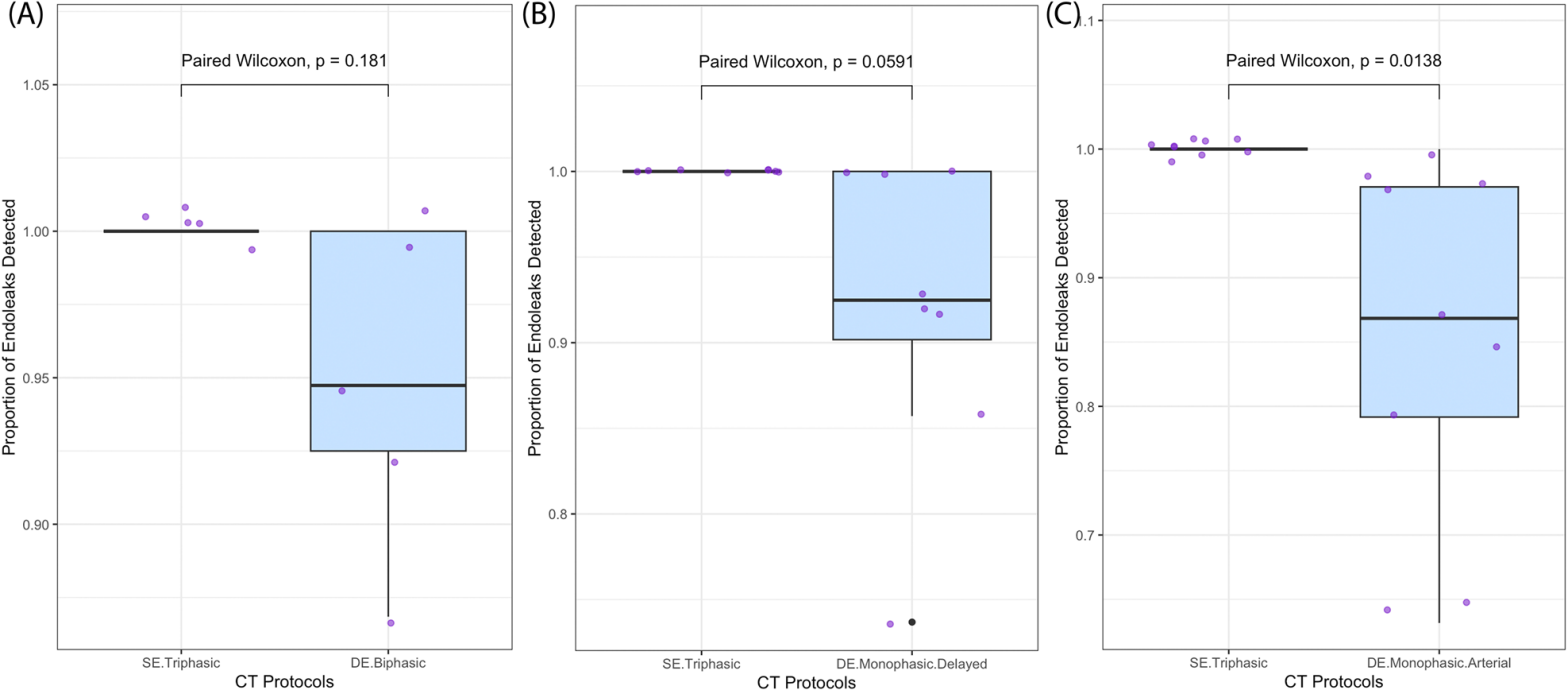
Boxplots showing the results of paired Wilcoxon tests comparing the proportion of endoleaks detected by the DE biphasic protocol (**A**), the DE monophasic delayed protocol (**B**) and the DE monophasic arterial protocol (**C**) against the reference standard SE triphasic CT. A *p* value of less than 0.05 was considered significant

The median percentage of endoleaks detected by DE biphasic protocol, DE delayed protocol and DE monophasic arterial protocol as compared against the reference standard triphasic SECT was 94.7% (IQR: 92.5%, 100.0%), 92.45 (IQR: 90.2%, 100.0%) and 86.8% (IQR: 79.2%, 97.1%), respectively. The paired Wilcoxon test showed no significant difference between the percentage of endoleaks detected by the DE biphasic and monophasic delayed protocol as compared to the reference standard SE triphasic protocol (*p* = 0.181 and 0.059, power = 83.2% and 62.4%, respectively) (Fig. 5). The paired Wilcoxon test also demonstrated significantly lower percentage of endoleak detected by the DE monophasic arterial protocol as compared to the reference standard (*p* = 0.014, power = 42.0%) (Fig. 5).

### Radiation dose

**Fig. 6:**
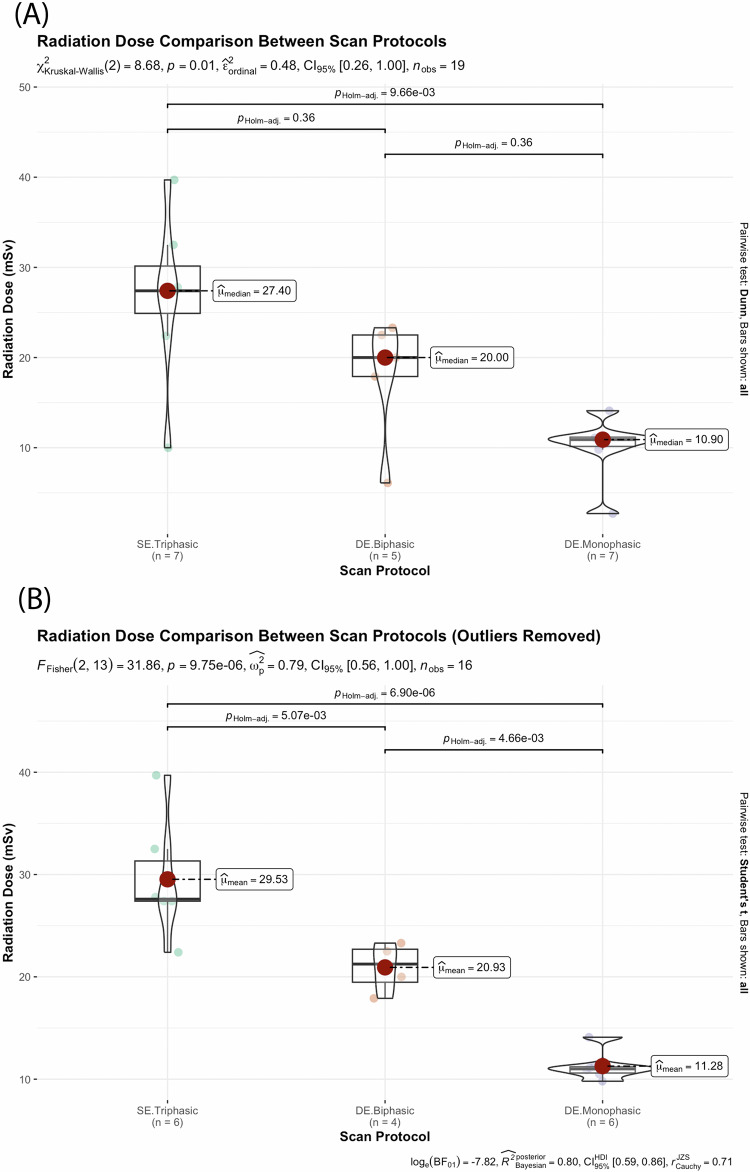
Statistical comparisons showing differences in radiation dose induced by the SE triphasic, DE biphasic and the DE monophasic CT with results of pair-wise comparisons reported. **A** Radiation dose data collected from seven studies were compared with the Kruskal–Wallis test due to the non-normal distribution of data. One outlier was identified under each group, resulting from one paper reporting abnormally low radiation dose for all three protocols. **B** After the removal of the outliers, a statistical comparison was conducted with one-way ANOVA due to the normal distribution of the datasets. A *p* value of less than 0.05 was considered significant

Seven studies reported the radiation dose (in mSv) of 579 CT scans [21–25, 28, 29]. The Kruskal–Wallis test using data collected from all 7 studies demonstrated an overall significant differences in radiation dose among the three CT protocols (*p* = 0.01), with the triphasic protocol displaying the highest median radiation dose at 27.4 (IQR: 24.90, 30.15) mSv, followed by DE biphasic protocol at 20.00 (IQR: 17.90, 22.50) mSv and the DE monophasic protocols at 10.9 (IQR: 10.15, 11.18) mSv (Fig. 6A). Pairwise comparisons demonstrated a significant difference in radiation dose between the SE triphasic and DE monophasic protocols (*p* = 0.0097) only (Fig. 6A). Three outliers were identified in total, with one found in each group (Fig. 6A). These results from the same study reporting radiation doses that were abnormally lower relative to other observations for all three CT protocols [28]. After removing these outliers, the datasets became normally distributed. Statistical comparisons using one-way ANOVA then revealed an overall significant difference in radiation dose among the three CT protocols (*p* < 0.001), as well as statistically significant differences with all pairwise comparisons (*p* < 0.001) (Fig. 6B). The mean radiation dose was highest for SE triphasic scans, at 29.53 ± 5.92 mSv. The DE biphasic protocol had the second highest mean radiation dose at 20.93 ± 2.46 mSv, while the DE monophasic protocol had the lowest mean radiation dose at 11.28 ± 2.48 mSv. Overall, reported studies showed DE biphasic and monophasic protocols can facilitate radiation reduction by approximately 29% and 62%, respectively, as compared to the triphasic SECT.

## Discussion

### Accuracy of DECT for endoleak detection post TEVAR/EVAR

This meta-analysis revealed that DECT had excellent accuracy in diagnosing endoleak. The results were robust in a sub-analysis of studies reporting diagnostic accuracy after EVAR only (Supplementary Fig. 1). This diagnostic accuracy is comparable, if not superior, to previously reported diagnostic accuracy parameters of the triphasic SECT, with sensitivity ranging from 58% to 96% and specificity estimated to be between 87% and 100% [36–39]. The meta-analysis revealed low levels of between-study heterogeneity, which could be attributed to multiple factors such as variations in study populations, CT scanners and timing of contrast-enhanced phases.

The quality and reliability of the VNC series have been the major concerns associated with DECT technology [40–42]. For the 803 DECT scans included in this review, no reported false positives or false negatives were attributed to poor image quality of VNC or inappropriate calcium subtraction by the VNC post-processing software.

Several unique DE reconstruction techniques, such as low kiloelectronvolt (keV) VMIs, iodine overlay and Hard Plaque imaging, were described by the studies included in this review (Supplementary Table S5). These additional reconstruction series were reported to have enhanced reader confidence [18, 24–27].

### Benefits and drawbacks

The most important benefit of adopting the DECT protocol for endoleak surveillance after endovascular aneurysm repair is the significantly lower radiation dose. The ability to obtain VNC from a DE contrast-enhanced scan means that the TNC phase may no longer be required. Depending on whether a biphasic or monophasic protocol is adopted, the DECT technique can achieve a dose reduction of 29% and 62%, respectively. Although the health risk associated with a single diagnostic CT scan is low, repeated scans can increase the lifelong risk of cancer [43]. A nationwide, population-based cohort study involving 24,645 patients from the UK revealed an increased risk of abdominal cancer after EVAR compared with open surgery, with the widespread use of CT surveillance and radiation exposure during fluoroscopy suggested as two possible causes [44]. As patients undergoing endovascular aortic repair are usually subjected to life-long imaging surveillance, a reduction in radiation by 29–62% is clinically significant.

One important drawback of DECT protocols is the obesity-related deterioration of image quality. This problem is more pronounced with DECT than with conventional SECT, as the unique technical features of the former can exacerbate truncation artefact, beam hardening and photon starvation when scanning patients of large body habitus [45, 46]. The truncation artefact is associated with the smaller field of view of DE systems, while beam hardening and photon starvation are caused by the inability of the photons of the low energy spectrum (typically at 80 kilovoltage peak) to penetrate the dense tissue [45, 46]. Two included studies reported false-positive and false-negative results caused by suboptimal image quality of DECT scans conducted on obese patients [21, 28], while three studies listed large body habitus as an exclusion criterion [24, 27, 29].

Another drawback of DECT is the false positive results caused by residual contrast material from the EVAR/TEVAR in scans performed immediately after endovascular procedures [21]. However, as the goal of surveillance scans is to detect endoleaks that potentially require monitoring, the occurrence of false positives is less critical compared to the risk posed by false negatives.

### Optimising the DECT protocol

Currently, there is no consensus on what phases of enhancement should be included in the DECT post-EVAR/TEVAR surveillance protocol. The VNC dataset is generally considered an indispensable component of the DECT surveillance protocol, as it depicts intraluminal calcifications without adding to the radiation dose [21, 47]. The delayed phase, on the other hand, is helpful for detecting low-flow endoleaks, especially a subset of type II endoleaks that are not visible on the arterial phase [22, 48, 49]. It has also been reported that VNC series derived from the delayed phase tend to have higher diagnostic quality and bear closer resemblance to TNC as compared to those derived from the arterial phase [40, 50]. The usefulness of the arterial phase, however, is controversial. Apart from having lower sensitivity to low-flow type II endoleaks as compared to the delayed phase, several studies also reported that all endoleaks visible on the arterial phase, including type I, II and III endoleaks, were subsequently visible on the delayed phase scan [22, 49]. Out of the ten included studies, only one reported a single case in which an endoleak was visible exclusively on the arterial phase scan [23].

These observations were supported by our analyses. The meta-analysis with a random effects model revealed a significant difference among the three DECT protocols in DOR, with the highest DOR value achieved by the biphasic protocol and the lowest observed with the monophasic arterial protocol (Fig. 4A, C). In addition, further analyses with paired Wilcoxon tests demonstrated that the percentage of endoleaks detected by the DE monophasic arterial protocol was significantly lower than that of the triphasic SECT (Fig. 5). These results suggest that the DECT performed with only an arterial phase scan may not be the best option for endoleak surveillance after endovascular aneurysm repair. However, when incorporated into the DECT biphasic protocol, the arterial phase may have played a supplementary role in improving diagnostic accuracy, allowing the biphasic protocol to achieve a higher DOR than that of the monophasic delayed protocol. The arterial phase might also be useful for detecting other peri-operative complications, such as arterial injury, pseudoaneurysms and puncture site complications [18, 49] and can be added to the scan protocol on a case-by-case basis when clinically indicated.

### Limitations

The major limitation of this meta-analysis was the small number of included studies, which reduced the statistical power of some of our analyses. Furthermore, all ten included studies were considered to be at high risk of bias and data on some outcomes, such as type IV and V endoleaks, medical history of participants, time from last EVAR and endoleak classification, were not reported in every study.

## Conclusion

Overall, this study suggests that DECT protocols can allow highly accurate endoleak detection with low radiation dose. For routine endoleak surveillance after endovascular repair, the DE biphasic protocol with both the arterial and delayed phase scans or the DE monophasic protocol with only a delayed phase scan is recommended, depending on the level of diagnostic accuracy required and the necessity for demonstrating peri-operative complications other than endoleaks.

## Supplementary information

ELECTRONIC SUPPLEMENTARY MATERIAL

## Abbreviations

AUC: Area under the curve
CI: Confidence interval
DE: Dual energy
DECT: Dual energy computed tomography
DOR: Diagnostic odds ratio
EVAR: Endovascular repair of aneurysm
IQR: Interquartile range
QUADAS-2: Quality assessment tool for diagnostic accuracy studies
SECT: Single energy CT
SROC: Summary receiver operating characteristics
TEVAR: Thoracic endovascular aneurysm repair
TNC: True non-contrast
VMIs: Virtual monoenergetic images
VNC: Virtual non-contrast
