# Supplementary material for: A meta-analysis of the diagnostic accuracy of dual-energy computed tomography for endoleak detection after endovascular aneurysm repair
Source: Eur Radiol. 2025 Jun 7;35(12):7951–62. doi: 10.1007/s00330-025-11717-8 (PMC12634740; doi:10.1007/s00330-025-11717-8)
Supplement: Supplementary file 1 — ELECTRONIC SUPPLEMENTARY MATERIAL [file 330_2025_11717_MOESM1_ESM.pdf]

A Meta-analysis of the Diagnostic Accuracy of Dual-Energy Computed Tomography for Endoleak Detection after Endovascular Aneurysm Repair

ELECTRONIC SUPPLEMENTARY MATERIAL

Supplementary Table S1. full search string used for database screening.

Database	Search string	Results
Pubmed	(("Dual"[All Fields] AND ("energie"[All Fields] OR "energies"[All Fields] OR "energy"[All Fields]) AND ("j comput tomogr"[Journal] OR "commun theory"[Journal] OR "child teenagers"[Journal] OR "cancer ther"[Journal] OR "ct"[All Fields])) OR ("Dual"[All Fields] AND ("energie"[All Fields] OR "energies"[All Fields] OR "energy"[All Fields]) AND ("tomography, x ray computed"[MeSH Terms] OR ("tomography"[All Fields] AND "x ray"[All Fields] AND "computed"[All Fields]) OR "x-ray computed tomography"[All Fields] OR ("computed"[All Fields] AND "tomography"[All Fields]) OR "computed tomography"[All Fields]))) AND ("endoleak"[MeSH Terms] OR "endoleak"[All Fields] OR "endoleaks"[All Fields])	31 studies
Scopus	(TITLE-ABS-KEY (dual AND energy AND ct) OR TITLE-ABS-KEY (dect) OR TITLE-ABS-KEY (dual AND energy AND computed AND tomography) AND TITLE-ABS-KEY (endoleak))	45 studies

Supplementary Table S2. Patient demographics and study type. Continuous variables are presented as mean ± standard deviation or median (interquartile range) as reported in individual studies.

Study		Study type	Sample size	No. of scans	Age (mean ± SD)	BMI (kg/m^2)/ weight (kg)	Male Sex (%)	Time from last EVAR/TEVAR	Clinical presentation at time of endovascular repair			Exclusion criteria
									AAA	TAA	TAAA	
Stolzmann et al., 2008		prospective	118	118	74 ± 8	27.0 ± 4.4	97 (82.2)	<1 week (14)<br>3 mo (26)<br>6 mo (27)<br>9 mo (22)<br>12 mo (41)	118	0	0	nephropathy, contrast allergy
Buffa et al., 2014		prospective	148	171	75 ± 6.5	28.3 ± 3.4	117 (79.1)	NR	148	0	0	Nephropathy, contrast allergy
Chandarana et al., 2008		prospective	22	24	76 (55-86)	NR	17 (68.2)	NR	22	0	0	Body habitus
Martin et al., 2017		retrospective	75	75	66.3 ± 11.7	25.7 ± 3.2	42 (56.0)	shortly after EVAR (mean, 4 days)<br>n= 27<br><br>follow up (mean, 2.5 years)<br>n = 48	75	0	0	Extravasation,<br>Motion artefacts<br>Flow rate of contrast injection <4
Müller-Wille et al., 2014		prospective	105	108	70 (42-86)	NR	83 (79.0)	shortly after repair (mean, 5 days)<br>n= 41<br><br>follow-up (mean, 3.3 years)<br>n= 68	75	24	6	NR
Maturen et al., 2011		retrospective	73	78	72 (56-93)	NR	53 (72.6)	NR	67	10	1	NR
Patino et al., 2019		prospective	41	41	77 (55-96)	85.5 ± 16.2	NR	NR	41	0	0	Nephropathy, contrast allergy,

												body habitus, without previous SECTA
Maturen et al., 2012		retrospective	39	39	75.6 (56-93)	NR	25 (64.1)	NR	19	20	0	Incomplete image capture
Flors et al., 2013		retrospective	48	74	66 (19-84)	NR	28 (58.3)	Mean, 524 days	0	48	0	Nephropathy, contrast allergy, body habitus
Melzig et al., 2024		prospective	75	75	73 ± 8.63	BMI <30 53 （70.7%） BMI ≥ 30 22 （29.3%）	63 (84)	NR	23	NR	NR	contrast allergy, pregnancy, high-grade cardiac insufficiency, cognitive impairment, inability to cooperate, without TEVAR or EVAR, Without previous SE triple phase CT

(BMI= body mass index; EVAR=endovascular repair of abdominal aortic aneurysm; TEVAR= Thoracic endovascular aortic repair; NR= Not reported; AAA= abdominal aortic aneurysm; TAA= thoracic aortic aneurysm; TAAA= thoracoabdominal aortic aneurysm; SECTA= single energy CT angiography)

Supplementary Table S3. Summary of CT acquisition details, including scanner models, timing of the contrast-enhanced phases, contrast protocols and the area covered in the scan.

study	Scanner model	DECT Acquisition Techniques	Scan protocol			Contrast protocol			Scan extend
			TNC	Arterial phase	Delay phase	Iodinated contrast (mL)	Saline chaser (mL)	Flow rate (mL/s)	
Stolzmann et al., 2008	Somatom Definition, Siemens	Dual-source dual energy	SE	SE	DE<br>70 s delay after initiation of contrast injection	120	30	4	From cardiac apex to greater trochanter for all three phases
Buffa et al., 2014	Somatom Definition, Siemens	Dual-source dual energy	SE	SE	DE<br>30 s after the arterial phase	80	40	4	From T12 to greater trochanter for all three phases
Chandarana et al., 2008	Somatom Definition, Siemens	Dual-source dual energy	SE	SE	DE<br>60 s after initiation of contrast injection	1.5 per kg body weight	NR	4	From T12 to pubic symphysis For all scans
Martin et al., 2017	Somatom Force, Siemens healthcare	Dual-source dual energy	SE	DE	SE<br>70 s after the start of contrast administration	80	NR	4	Not specified
Müller-Wille et al., 2014	Somatom Definition Flash, Siemens, Forchheim, Germany	Dual-source dual energy	SE	SE	DE<br>70 s after the start of arterial phase	90	NR	4	TNC and DE delay phase covered the length of stent graft<br>Arterial phase covered the whole aorta
Maturen et al., 2011	GE HD-750 (GE Healthcare, Milwaukee, Wis)	Fast-switching kVp	SE	DE	DE<br>60 s after arterial phase	120	50	4	TNC: NR<br>Arterial phase: abdomen and pelvis (± chest)<br>Delay phase: area of stent graft

Patino et al., 2019	**Reference standard:** LightSpeed/VCT GE healthcare; Somatom Definition Edge, Siemens Healthcare	SE mode	SE	SE	SE<br>120 s after initiating contrast injection	80-90	40	3.5	Abdomen and pelvis for all phases
	**Index test:** Discovery CT 750HD, GE Healthcare	Rapid-kV-switching	SE	DE	DE<br>60 s after initiating contrast injection	50-60	40	3[8] or 2.8[1]	Abdomen and pelvis for all phases
Maturen, et al 2012	Discovery CT 750HD, GE Healthcare	Rapid-kV-switching	SE	DE	DE<br>60 s after initiating contrast injection	120	50	4	TNC: NR<br>Arterial: entire abdomen and pelvis ± chest<br>delay: through region of endograft
Flors et al, 2013	Somatom Definition, Siemens Healthcare	Dual-source Dual energy	SE	SE	DE<br>300 s after initiating contrast injection	100	25	4	TNC and delay phase: 3 cm above the stent to 1 cm below the stent<br>Arterial phase: pulmonary apex to lowest point of diaphragm
Melzig et al., 2024	Somatom Definition Flash, Siemens Healthcare	Dual source dual energy	SE	SE	SE<br>Exact timing of delay phase not specified	90	40	4	46 of 75 (61.3%) covered entire aorta<br>23 of 75 (30.7%) covered only the abdominal aorta
			NR	SE	DE<br>Exact timing not specified	54(BMI <30)<br>or<br>60 (BMI ≥30)	30	3.7 (BMI <30)<br>4.1 (BMI ≥30)	

(DECT= dual energy CT; TNC= true non-contrast phase; DE= dual energy; SE= single energy; NR= not reported; BMI=body mass index)

Supplementary Table S4. A summary of study design detailing the standard of reference, index tests, reading sessions, and blinding of readers adopted by individual studies.

Study	Reference standard	Index test – DE biphasic	Index test - DE monophasic	No. of radiologists	Image Interpretation	Blinded?
Stolzmann et al., 2008	Reconstructed “SE” triphasic protocol	VNC_{delay} + SE arterial phase + DE delay phase + relevant reconstructions	VNC_{delay} + DE delay + relevant reconstructions	2	Session 1: reference standard<br>Session 2: biphasic index test<br>Session 3: monophasic index test<br>With a 1 – week gap between each session	Yes
Buffa et al., 2014	Reconstructed “SE” triphasic protocol	N/A	VNC_{delay} + DE delay + relevant reconstructions	3	2 radiologists reviewed reference standard.<br>a third radiologist reviewed index test independently.	NR
Chandarana et al., 2008	Reconstructed “SE” triphasic protocol	N/A	VNC_{delay} + DE delay + relevant reconstructions	3	2 radiologists reviewed index test; a different radiologist reviewed standard of reference	NR
Martin et al., 2017	Reconstructed “SE” triphasic protocol	N/A	TNC + DE Arterial + relevant reconstructions	2	Session 1: reference standard<br>Session 2: index test<br>With a 2-week gap in between	Yes
Müller-Wille et al., 2014	Reconstructed “SE” triphasic protocol	N/A	(1) VNC_{art} + DE arterial<br>(2) VNC_{delay} + DE delay<br>Both with relevant reconstructions	3	1 radiologist reviewed reference standard series independently; 2 other radiologists reviewed the index tests images	Yes
Maturen et al., 2011	Reconstructed “SE” triphasic protocol	N/A	(1) VNC_{art} + DE arterial<br>(2) VNC_{delay} + DE delay<br><br>Both with relevant reconstructions	2	Session 1: index test (1)<br>Session 2: index test (2)	Yes

Patino et al., 2019	Reconstructed "SE" triphasic protocol	N/A	50 keV and 40 keV VMIs of the DE arterial phase	3	2 radiologists reviewed the index tests; a third radiologists interpreted reference standard	Yes
Maturen, et al 2012	Reconstructed "SE" triphasic protocol	TNC + DE arterial phase + DE delay phase with 55 keV and 75 keV VMIs for both DE phases	TNC + 55 keV and 75 keV VMIs of DE arterial<br><br>TNC + 55 keV and 75 keV VMIs of DE delay	2	Session 1: TNC+ 55 keV DE arterial images<br>Session 2: TNC+ 75 keV DE arterial images<br>With a 2-week gap between sessions	Yes
Flors et al, 2013	Reconstructed "SE" triphasic protocol	VNC_{delay} + SE arterial phase + DE delay phase + relevant reconstructions	VNC_{delay} + DE delay + relevant reconstructions	2	Session 1: reference standard<br>Session 2: DE monophasic index test<br>Session 3: DE biphasic index test	Yes
Melzig et al., 2024	a previous true SE triphasic scan	VNC_{delay} + SE arterial phase + DE delay phase + relevant reconstructions	N/A	2	2 readers independently assessed the DE biphasic protocol	No

(SE=single energy; DE= Dual energy; VNC= virtual non-contrast; N/A= not assessed; NR= not reported)

Supplementary Table S5. The types and numbers of DECT image reconstructions conducted by individual studies.

reconstructions / Study	VNC[1]	Weighted average dataset[2]	Material decomposition images (Iodine)	Material decomposition images (Calcium)	VMIs[3]	Types of reconstructions performed
Stolzmann et al., 2008	Yes	Yes	N/A[7]	N/A	N/A	2
Buffa et al., 2014	Yes	Yes	N/A	N/A	N/A	2
Chandarana et al., 2008	Yes	Yes	N/A	N/A	N/A	2
Martin et al., 2017	N/A	Yes	N/A	N/A	40 – 100 keV traditional and noise reduced VMI series with 10 keV intervals	2
Müller-Wille et al., 2014	Yes	Yes	Iodine map	Hard Plaque Imaging (HPI) applications[5]	N/A	4
Maturen et al., 2011	Yes	Likely[6]	N/A	N/A	N/A	2[6]
Patino et al., 2019	N/A	Likely[6]	N/A	N/A	40 keV and 50 keV VMIs	2[6]
Maturen, et al 2012	N/A	N/A	N/A	N/A	55 keV and 75 keV	1
Flors et al, 2013	Yes	Yes	Iodine overlay display[4]	N/A	NA	3
Melzig et al., 2024	Yes	Likely[6]	N/A	N/A	N/A	2[6]

1. Virtual non-contrast: a “non-contrast” dataset created by subtracting iodine signal from a contrast-enhanced DE dataset.
2. A fused image dataset that combines the characters of the low- and high- voltage acquisitions by assigning weighting factors. This results in images simulating standard 120 kV SE images.
3. Virtual monoenergetic images: reconstructed dataset that simulate CT images obtained with monochromatic x-rays of a specific photon energy.
4. Images containing both the grey-scale structural information and a coloured overlay representing iodine
5. An application that allows for the differential display of calcium (red coded) and iodine (blue coded) on the same image dataset.
6. The study did not mention the creation of a weighted average dataset. However, the DE delay images provided as representative examples resembles more of a weighted average dataset rather than the untreated 80 kVp or 120 kVp dataset.
7. N/A: not assessed; reconstruction not performed.

Supplementary Figure 1. Sub-analysis of diagnostic accuracy data of DECT protocols collected from EVAR-only studies (n=5) with random effects model. The forest plots displayed the aggregated means of sensitivity (A), specificity (B) and DOR (C) reported with 95% confidence intervals. The SROC curve (D) was plotted with 95% confidence interval region and data points representing sensitivity and false positive rate (1-specificity) generated from each individual studies.

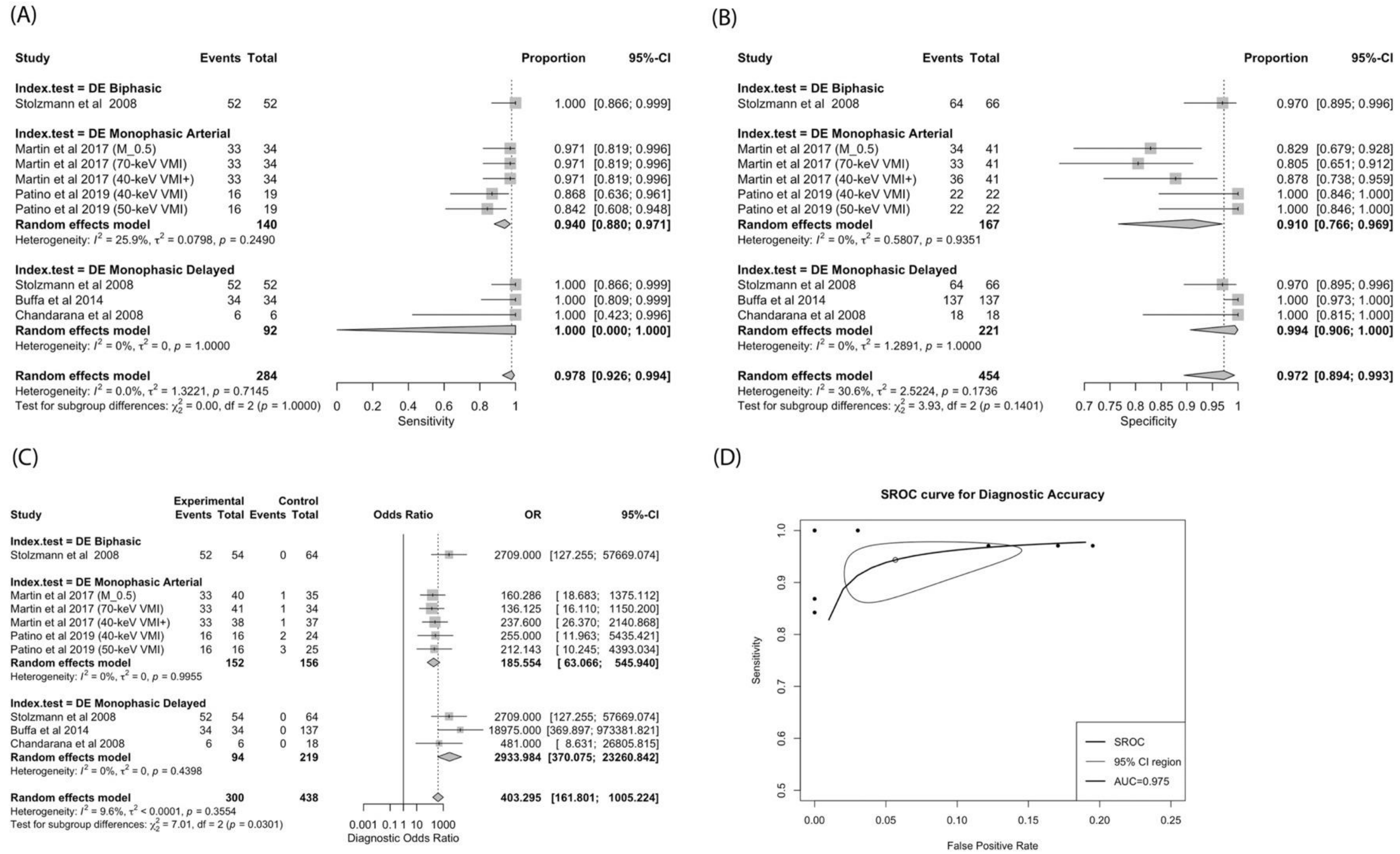

Eur Radiol (2025) Wen CX, Thanigaimani S, Brennan S, Moxon J, Golledge J.
